# Supplementary figures and images for: A unifying model that explains the origins of human inverted copy number variants
Source: PLoS Genet. 2024 Jan 4;20(1):e1011091. doi: 10.1371/journal.pgen.1011091 (PMC10766186; doi:10.1371/journal.pgen.1011091)

S1\_Fig

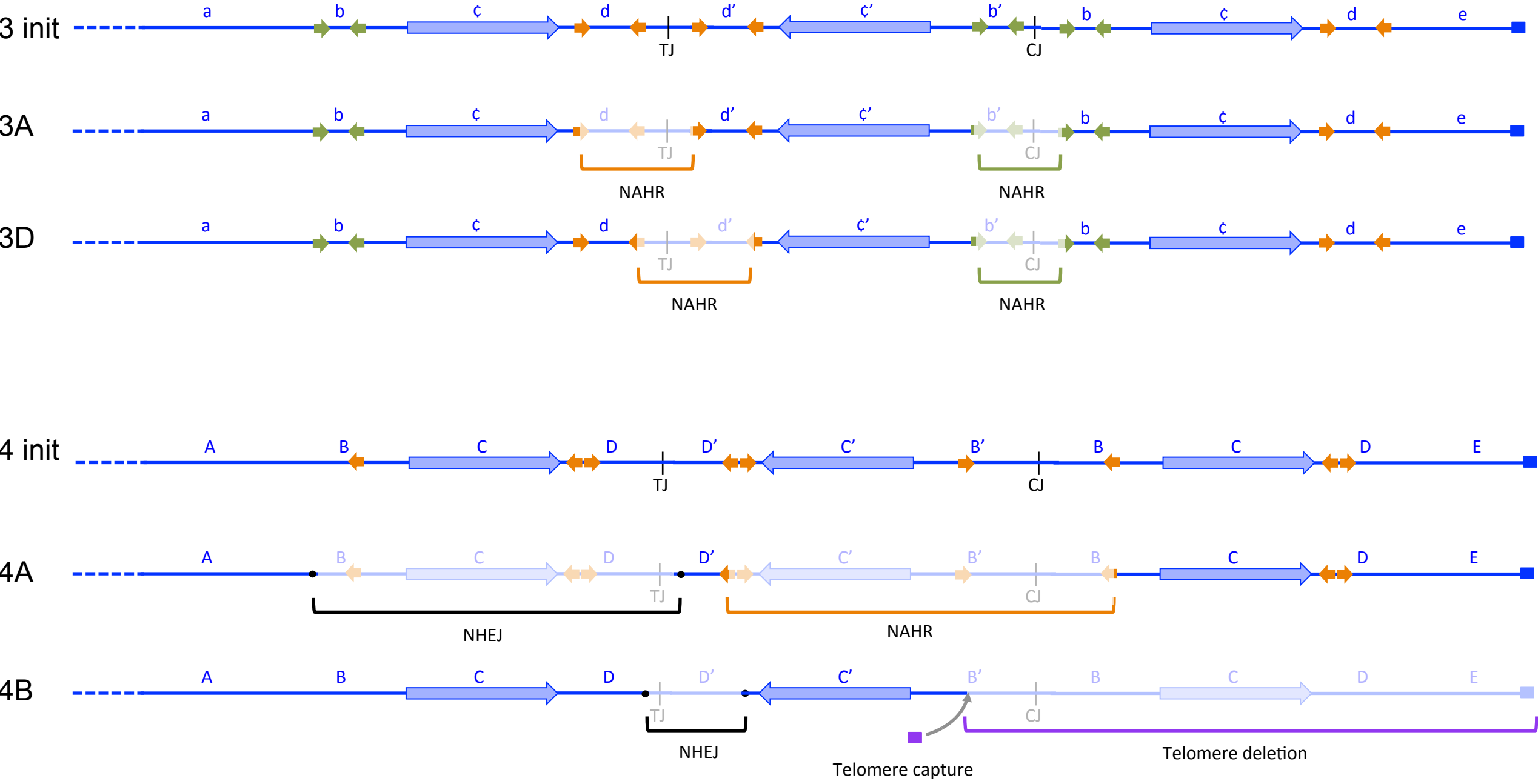

Supplement: S1 Fig — 3init, 3A and 3D refer to images from Fig 3. Init = inverted triplication before rearrangement. 4init, 4A and 4B refer to images from Fig 4. Init = inverted triplication before rearrangement. Brackets indicate rearrangement junctions created by NAHR, NHEJ, or MMBIR. The grayed-out regions indicate the regions of the inverted triplication that are deleted during the secondary rearrangements. (PDF) [file pgen.1011091.s001.pdf]
